# VDAC2 and the BCL-2 family of proteins

**DOI:** 10.1042/BST20210753

**Published:** 2021-12-16

**Authors:** Zheng Yuan, Grant Dewson, Peter E. Czabotar, Richard W. Birkinshaw

**Affiliations:** 1 The Walter and Eliza Hall Institute of Medical Research, Parkville, Victoria 3052, Australia; 2 Department of Medical Biology, The University of Melbourne, Parkville, Victoria 3010, Australia

**Keywords:** apoptosis, BAK, BAX, BCL-2 family proteins, VDAC2, voltage-gated channels

## Abstract

The BCL-2 protein family govern whether a cell dies or survives by controlling mitochondrial apoptosis. As dysregulation of mitochondrial apoptosis is a common feature of cancer cells, targeting protein–protein interactions within the BCL-2 protein family is a key strategy to seize control of apoptosis and provide favourable outcomes for cancer patients. Non-BCL-2 family proteins are emerging as novel regulators of apoptosis and are potential drug targets. Voltage dependent anion channel 2 (VDAC2) can regulate apoptosis. However, it is unclear how this occurs at the molecular level, with conflicting evidence in the literature for its role in regulating the BCL-2 effector proteins, BAK and BAX. Notably, VDAC2 is required for efficient BAX-mediated apoptosis, but conversely inhibits BAK-mediated apoptosis. This review focuses on the role of VDAC2 in apoptosis, discussing the current knowledge of the interaction between VDAC2 and BCL-2 family proteins and the recent development of an apoptosis inhibitor that targets the VDAC2–BAK interaction.

## Introduction

Apoptosis is a major form of programmed cell death that clears potentially dangerous cells or cells that are no longer needed. Hence, apoptosis is important to maintain our health, as many cells in our body must die for embryonic development, maintenance of normal tissue homeostasis and a regulated immune response to infection. Insufficient apoptosis has been implicated in cancer [1,2] and autoimmunity [3], while excessive apoptosis is often associated with neurodegenerative disease [4,5]. The BCL-2 protein family tightly regulates mitochondrial apoptosis. The family is sub-divided by apoptotic phenotype into pro-survival (e.g. BCL-X_L_, BCL-2 and MCL-1) and pro-apoptotic proteins (e.g. BIM, BAK and BAX) [6]. The subtle balance between the two groups determines the cell life/death decision.

The pro-apoptotic effector proteins BAK and BAX are executioners of the apoptotic pathway; their conformational changes and oligomerisation in response to apoptotic stressors cause mitochondrial outer membrane permeabilisation (MOMP) [6]. This pivotal step releases cytochrome *c* and other apoptogenic factors from mitochondria to trigger the activation of cellular proteases and caspases to fully commit the cell to apoptosis [7]. The BCL-2 protein family share sequence homology in up to four conserved motifs called the BCL-2 homology (BH) domains, of which BAK and BAX contain all four. Structurally, BAK and BAX consist of nine α-helices (α1–9), including a transmembrane (TM) domain (α9), and exhibit a typical BCL-2 fold: seven α-helices folding around the central hydrophobic α5-helix [8,9]. A key feature of this fold is a hydrophobic surface groove formed by α-helices 2 to 5. This surface groove is key for their interactions with the BH3 domains of the pro-apoptotic proteins (e.g. BIM or another BAK/BAX protein) [10–12]. In addition, there are reports of a second rear pocket between α-helices 1 and 6 where BH3 peptides can also interact [13–16]. Binding of a BH3 domain to either site triggers activation and unfolding of the BAK and BAX BCL-2 fold. This unfolding event transitions BAK and BAX from their inert monomers into activated homo-dimers that then oligomerise and cause MOMP [10,11,17].

**Figure 1.**
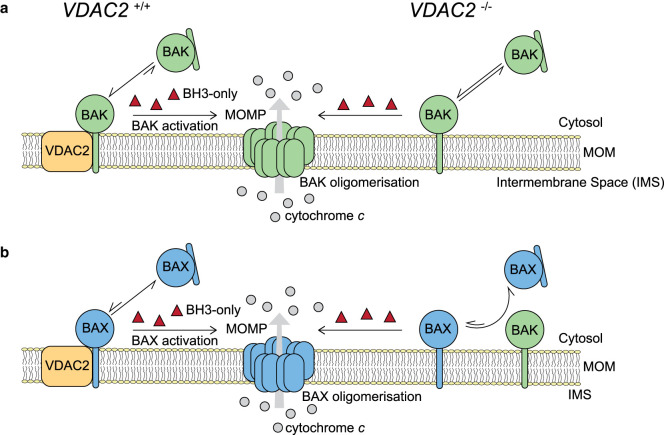
VDAC2 is important for BAK and BAX targeting mitochondria. The pro-apoptotic effectors BAK and BAX interact with VDAC2 on the mitochondrial outer membrane (MOM) to enable membrane recruitment [24]. Induction of apoptosis by up-regulation of BH3-only proteins activates BAK and BAX regardless of their starting subcellular localisation, and they dissociate from VDAC2 to cause MOM permeabilisation (MOMP) and cytochrome *c* release from the inter membrane space (IMS) [24,25]. (**a**) BAK can still migrate to the mitochondria in a VDAC2-deficient (*VDAC2^−/−^*) setting [23–26]. (**b**) However, in the absence of VDAC2, BAX becomes dependent on BAK for its mitochondrial targeting [24,25]. Arrows indicate movement between BAK and BAX localisation and conformation, with arrow length indicating the preference for localisation (smaller is less prominent).

BAK and BAX have been suggested to function redundantly [18,19], despite potential differences in the levels of tissue expression and subcellular localisation (Figure 1) [20]. BAX is in a dynamic equilibrium between the cytosol and mitochondria, [21] and is predominantly found in the cytosol in its inactive state, as its α9-helix transmembrane (TM) domain is sequestered in its hydrophobic groove (Figure 1) [8]. In contrast, BAK is constitutively localised to mitochondria via its TM domain [22] and where it forms complexes with VDAC2 (Figure 1) [23]. In this review, we summarise the roles of VDAC2 in apoptosis and specifically discuss its interactions with BAK and BAX and the consequences for apoptotic progression.

## VDAC2 in apoptosis

Voltage Dependent Anion Channels (VDACs), also known as mitochondrial porins, are a family of β-barrel membrane proteins that are the most abundant proteins in the mitochondrial outer membrane (MOM) [27]. VDACs allow passage of both negatively and positively charged ions [28–30], NADH [31], ATP/ADP [32] and other metabolites [33] across the MOM. Three VDAC isoforms (1, 2 and 3) are found in mammals, which show high sequence similarity and overlapping tissue expression [30,34]. They share responsibility in metabolite flux function and have been implicated in many pathways including glycolysis [35] and release of reactive oxygen species (ROS) [36,37]. Among the 3 VDAC isoforms, VDAC2 has drawn considerable interest as VDAC1 or VDAC3 knockout mice displayed no overt to mild phenotypes [38–40], while VDAC2-deficient embryos died during development [23,25]. This embryonic lethality was proposed to arise from its role in regulating apoptosis [41].

VDAC2 was originally thought to form part of the mitochondrial permeability transition pore (PTP) complex which allowed the release of cytochrome c during apoptosis [42]. This hypothesis was based on VDAC2's channel function, its abundance in the MOM and its interactions with pro-survival proteins BCL-2 [43,44] and BCL-X_L_ [45,46]. Several models have been proposed [45,47] and well-reviewed [48]. While evidence for a role of VDAC in MOMP accumulated, genetic studies from Baines and colleagues [49] refuted this hypothesis. In cells lacking all three isoforms of VDAC mitochondrial permeability transition and also apoptosis were unaffected [49]. This indicated that VDAC proteins are dispensable for apoptosis and that cytochrome *c* was released by another mechanism, notably via activation and oligomerisation of BAX and BAK [6].

As VDAC2 was not required for apoptosis, the field shifted direction focusing on its interactions with the pro-apoptotic proteins BAK and BAX. This began with Cheng and colleagues investigating potential binding partners of BAK on the MOM [23]. Using chemical crosslinking methods, they found that mitochondrial BAK could cross-link with VDAC2, but not VDAC1 or 3. This VDAC2–BAK complex was disrupted when apoptosis was initiated, and they found that overexpression of VDAC2 inhibited BAK-mediated apoptosis. Furthermore, mouse embryonic fibroblasts (MEFs) lacking VDAC2 were more sensitive to apoptosis induced by the BH3-only protein BID. This suggests that VDAC2 inhibits BAK-mediated apoptosis by sequestering BAK in its inactive form. BAK was subsequently reported to dissociate from the MOM in VDAC2-deficient cells and that tBID was required to recruit BAK to the MOM [24]. Hence, in VDAC2-deficient cells the mitochondria-associated pool of BAK does not promote constitutive apoptosis, but requires additional stimuli to recruit BAK to mitochondria for effective cytochrome *c* release [24]. Therefore, VDAC2 plays a role both in recruiting BAK to the MOM and in inhibiting its activation (Figure 1).

While BAK-mediated apoptosis can still occur in the absence of VDAC2, contrastingly BAX requires VDAC2 to mediate apoptosis [24,25]. This is due to a requirement of BAX for VDAC2 (or BAK) at mitochondria for efficient mitochondrial localisation (Figure 1). Furthermore, in cells where apoptosis is solely mediated by BAX, deletion of VDAC2 could provide long-term protection against induction of apoptosis by small molecule ‘BH3-mimetics’ apoptotic agents [25]. Hence, VDAC2 has contrasting roles in regulating BAX and BAK-mediated apoptosis.

The localisation of BAK and BAX to the MOM is counterbalanced by retrotranslocation of both BAK and BAX to the cyotosol, potentially coordinated by the pro-survival protein BCL-X_L_ [50,51], although a mechanism independent of BCL-2 proteins has also been proposed [21]. Retrotranslocation is more prominent for BAX, helping to maintain its residence in the cytosol in the absence of apoptotic stimuli. For BAK the retrotranslocation rate is low, which is consistent with BAKs predominant localisation at the MOM [51]. The interaction between VDAC2 and BAK is proposed to prevent BAK retrotranslocation by BCL-X_L_ [52]. This is consistent with the observations that BAK redistributes to the cytosol in VDAC2 deficient cells [24].

Beyond protein–protein interactions, another consideration is the effect of lipid on BAK, BAX and VDAC and their oligomerisation. All three proteins perform their critical function in a lipid membrane. Lipids can regulate VDAC1 oligomerisation, with cardiolipin disrupting and phosphatidylglycerol promoting VDAC1 oligomers [53]. Interestingly, cardiolipin is also required for efficient tBid recruitment to the MOM to promote BAX homo-oligomerisation during apoptosis [54–56]. In addition, lipids have been shown to regulate BAK/BAX activation and stabilise BAK oligomers [57–59]. However, roles for cardiolipin and other lipids in regulating VDAC2 interactions with BAK and BAX are not currently established.

## Unmasking the VDAC2–BAK binding interface

BAK integrates into the VDAC2–BAK complex in its inactive form [23]. The C-terminal TM domain (α9-helix) and preceding hydrophilic segment (mBAK 186–209, referred to as ‘BAK C-terminus’ hereafter) play an essential role in both targeting BAK to mitochondria and its interaction with VDAC2. This was demonstrated by replacing the BAK C-terminus with that of Fis1 (BAK-Fis1^TM^), a protein that like BAK, can anchor to the MOM with its C-terminal mitochondrial targeting sequence [26]. This chimeric BAK-Fis1^TM^ protein maintained mitochondrial localisation, but failed to assemble into the VDAC2 complex. Conversely, when GFP is fused with the BAK C-terminus, this GFP-BAK^TM^ molecule targets mitochondria, but is again unable to recognise VDAC2 [60]. These data show that the BAK C-terminus is critical for VDAC2 interactions, but also indicate that additional regions in the BAK α1–8 core domain are required to stabilise the VDAC2–BAK interaction.

**Figure 2.**
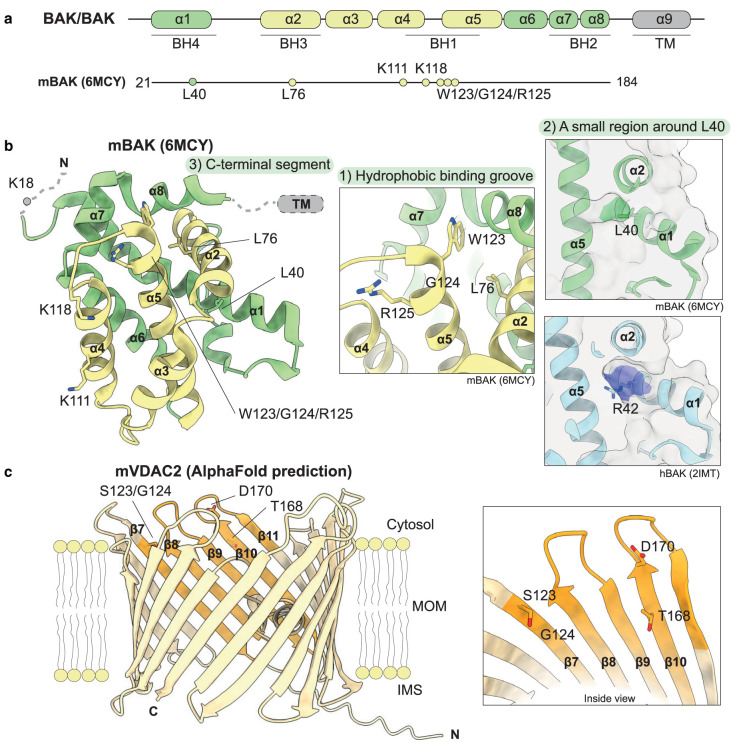
Potential interface on BAK and VDAC2 involved in VDAC2–BAK interaction. (**a**) Diagram of pro-apoptotic effector proteins BAX and BAK with proposed VDAC2 interacting residues on mouse BAK (mBAK). (**b**) The crystal structure of mBAK (PDB: 6MCY) [61] is shown in cartoon representation with α-helices 2 to 5 forming the hydrophobic surface groove shown in yellow; residues implicated in the VDAC2–BAK interaction are shown as sticks. The proposed interacting residues on BAK can be classified into three hot spots: (1) the BAK canonical hydrophobic binding grove formed by α-helices 2–5; (2) a small region behind the canonical binding groove centred around L40 in mBAK (green) which is equivalent to R42 in human BAK (hBAK, cyan, PDB: 2IMT) [9]; (3) the BAK TM domain (not resolved in this structure) and the C-terminus of α8. (**c**) AlphaFold2 predicted model of mVDAC2 (UniProt: Q60930) is shown in yellow cartoon with β7–10 highlighted in orange [62,63]. The region on VDAC2 involved in VDAC2–BAK interaction has been mapped to its β7–10 region, where T168 and D170 are considered crucial while S123 and G124 are also relevant. They all locate to the cytosol-orientated region of VDAC2, facing towards the inside of the pore.

Chemical cross-linking studies in MEFs show that BAK can cross-link with VDAC2 using the primary amine cross-linker disuccinimidyl suberate (DSS) [23]. Mouse BAK contains only four primary amine groups (-NH_2_) that are available for this reaction located at the N-terminus, in the N-terminal flexible region (K11), or in the α4-helix (K111 and K118), which indicates the potential interfaces of BAK that are in proximity to VDAC2 (Figure 2). Mutagenesis studies suggest the VDAC2–BAK interaction is lost with the BAK BH3 domain mutant L76E and reduced with BAK BH1 domain triple mutant W123A/G124E/R125A (Figure 2) [23]. This implies that BAK interacts with VDAC2 through its BH1 and BH3 domains, which are both part of the BAK hydrophobic BH3 binding groove (Figure 2).

VDAC2 is an integral β-barrel membrane protein consisting of 19 β-strands with an N-terminal α-helix that resides within the pore (Figure 2) [64]. There is currently no experimentally determined structure available for mammalian VDAC2; however, structures exist for human/mouse VDAC1 chimeras [65–67] and zebrafish VDAC2 [64], which are normally used as models for the mammalian orthologues. There are now high confidence molecular models publicly available thanks to a collaboration between the European Bioinformatics Institute (EBI) and DeepMind's AlphaFold2 structure prediction pipeline (Figure 2) [62,63]. Despite 75% sequence identity between VDAC1 and VDAC2, their ability to bind BAK has diverged, providing an opportunity to unmask the BAK binding interface on VDAC2. Gain-of-function studies with VDAC1/2 chimeras show that β-strands 7–11 are required for BAK to bind VDAC2 [30]. Several residues in this region including T168 and D170 have been identified critical for BAK binding, while S123 and G124 are also involved (Figure 2) [30].

These studies have narrowed down the regions on BAK and VDAC2 that interact; however, they do not explain exactly how VDAC2 and BAK interface. This detail is required to understand how other proteins, or potentially small molecules, could disrupt or stabilise the complex to alter apoptosis.

## The nature of the VDAC2–BAK complex

While evidence of the VDAC2–BAK interaction accumulates, the role of this protein–protein interaction has gradually become unmasked.

As discussed above, VDAC2 is suggested to play an important role in targeting BAK to mitochondria [26], though not essential, as BAK can still reach the mitochondria in VDAC2-deficient cells despite being primarily localised to the cytosol [25,26]. This was further demonstrated by an *in vitro* protein import assay into mitochondria. The level of *in vitro*-imported BAK was compromised by the knockdown of VDAC2 but not VDAC1 [60]. Conversely, overexpression of VDAC2, but not VDAC1, increased mitochondrial accumulation of BAK.

The VDAC2–BAK complex can be isolated from non-apoptotic mitochondria by digitonin extraction, with an apparent and approximate mass of 400 kDa according to Blue Native gel electrophoresis [24,26]. Quantitative mass spectrometry data confirmed the presence of VDAC2 and BAK in this complex, as well as VDAC1 and VDAC3 [25]. Although deletion of VDAC1 or VDAC3 disrupted this 400 kDa mitochondrial VDAC2–BAK complex, VDAC1 and VDAC3 are not thought to interact with BAK directly and only VDAC2 is required for efficient BAK localisation to mitochondria [60]. When apoptosis is initiated, the larger VDAC2–BAK complex is disrupted and BAK homo-oligomers are formed, suggesting that BAK dissociates from VDAC2 upon apoptosis [24]. Furthermore, cytochrome *c* release in mitochondria lacking VDAC2 is indeed faster than in mitochondria from wild-type MEFs, suggesting a potential restraining role for VDAC2 in regulating BAK apoptotic function [26].

## Targeting the VDAC2–BAK interaction

The importance of the VDAC2–BAK interaction is highlighted by a small molecule developed by van Delft and colleagues [61]. This molecule, WEHI-9625, is a selective mouse BAK inhibitor that blocks apoptosis before MOMP and affords cells long-term protection in response to apoptotic stress in clonogenic assays [61]. While activating apoptosis with BH3 mimetic small molecules is clinically validated for cancer treatment [68], the development of WEHI-9625 paves the way towards small molecules that block unwanted apoptosis. WEHI-9625 acts to prevent BAK function by stabilising the VDAC2–BAK interaction, hence it prevents disassociation of the VDAC2–BAK complex in cells under apoptotic stress. This highlights the key role VDAC2 plays in regulating apoptosis and reveals the physiological consequence of stabilising VDAC2–BAK interaction.

The defined binding interface of WEHI-9625 remains unknown, however, it is appreciated that VDAC2 is required for WEHI-9625 to function. This was demonstrated by a pull-down assay using a chemical probe analogue of WEHI-9625 with UV photocross-linking in MEFs [61]. While both VDAC1 and 2 were significantly enriched, only VDAC2 was found to be essential for WEHI-9625 function in cells. In addition, gain-of-function studies using VDAC1/2 chimeras suggests that some residues in the cytosol facing segment of VDAC2 β7–10 are important for WEHI-9625 function [61]. This data aligns with previous studies indicating the essential role of VDAC2 β7–11 in targeting BAK to mitochondria and assembly of the VDAC2–BAK complex [30].

Curiously, WEHI-9625 only inhibits mouse BAK-mediated apoptosis and is completely inactive against human BAK. Given the marked divergence of WEHI-9625's ability to block mBAK and hBAK function, a series of m/hBAK chimeras were generated for activity studies. This narrowed down the region of BAK involved in the interface to two regions. The first involved several residues at the C-terminus of α8 (F181, R182, R183, D184 and T188 based on mBAK) that are in proximity to the transmembrane domain. Part of this region is poorly defined in BAK experimental structures [61] and likely flexible, making it difficult to understand how this region contributes to compound binding. The second region locates to the middle of α1 (L40), which is involved in a defined pocket behind the canonical hydrophobic groove. In the case of hBAK, this L40 is replaced by R42 which points into, and changes the nature of, the groove in this region [12]. Neither of these regions have been implicated in VDAC2–BAK interaction previously. Combined with the aforementioned crosslinking data [23], the regions of BAK that are implicated in the VDAC2–BAK interaction are revealed as, (1) a small region behind the canonical binding groove centred around L40; (2) the BH1 and BH3 domains within the canonical hydrophobic binding groove; and (3) the C-terminal segment which is either in proximity to, or embedded in, the membrane.

## Conclusions

VDAC2 has emerged as a non-BCL-2 family protein that regulates apoptosis. Although the exact molecular mechanism behind this is not yet completely understood, it is now appreciated that VDAC2 differentially controls BAK and BAX apoptotic activity. VDAC2 is important for both BAX and BAK targeting to mitochondria where they execute apoptosis. BAK forms a distinct complex with VDAC2 on the MOM, which it dissociates from, in response to apoptotic signals [24,25]. The interaction between BAK and VDAC2 is a potential drug target, now highlighted by the development of WEHI-9625, a selective mBAK inhibitor. However, there are currently no experimental structures available for mammalian VDAC2 or full-length BAK with its TM domain. Instead, the VDAC2–BAK interface has been mapped by chemical cross-linking and mutagenesis approaches. Unfortunately, these studies lack the information required to understand the mechanism by which WEHI-9625 prevents dissociation of BAK from VDAC2. The VDAC2–BAK interface can be narrowed down to the single β7–10 region on VDAC2 [30], but the proposed interaction sites on BAK are separated, where its C-terminal segment, TM domain [26,60] and residues in proximity to the hydrophobic groove [23,61] are implicated. Mapping the VDAC2–BAK interaction at the molecular level will be pivotal for understanding the mechanism of WEHI-9625. Understanding this mechanism is key for developing similar inhibitors for human cells and assessing their potential to treat human disease.

## Perspectives

- The last twenty years has shown VDAC2 plays a role in apoptosis which is dependent on BAK and BAX.
- VDAC2 facilitates the import of both BAK and BAX into the mitochondrial outer membrane, where it restrains BAK activation.
- A small molecule (WEHI-9625) protects against apoptosis and is dependent on both VDAC2 and mouse BAK but does not affect the human BAK orthologue. Understanding this selectivity will inform if apoptosis can be inhibited by a similar mechanism in human cells and can guide new avenues for therapy.

## Open Access Statement

Open access for this article was enabled by the participation of The Walter and Eliza Hall Institute of Medical Research, in an all-inclusive *Read & Publish* pilot with Portland Press and the Biochemical Society under a transformative agreement with CAUL.

## Abbreviations

BH: BCL-2 homology
DSS: disuccinimidyl suberate
EBI: European Bioinformatics Institute
IMS: inter membrane space
MEF: mouse embryonic fibroblast
MOM: mitochondrial outer membrane
MOMP: mitochondrial outer membrane permeabilisation
PTP: permeability transition pore
ROS: reactive oxygen species
TM: transmembrane
VDAC: Voltage dependent anion channel 2
